# Phase II study of weekly vinorelbine and 24-h infusion of high-dose 5-fluorouracil plus leucovorin as first-line treatment of advanced breast cancer

**DOI:** 10.1038/sj.bjc.6602469

**Published:** 2005-03-15

**Authors:** K H Yeh, Y S Lu, C H Hsu, J F Lin, H J Chao, T C Huang, C Y Chung, C S Chang, C H Yang, A L Cheng

**Affiliations:** 1 National Taiwan University Hospital, Taiwan; 2 National Taiwan University College of Medicine, Taiwan; 3 Far Eastern Memorial Hospital, Taiwan; 4 Changhua Christian Hospital, Taiwan; 5 National Health Research Institutes, Taiwan

**Keywords:** weekly vinorelbine, high-dose 5-FU and leucovorin, breast cancer

## Abstract

We prospectively investigated the efficacy and safety of combining weekly vinorelbine (VNB) with weekly 24-h infusion of high-dose 5-fluorouracil (5-FU) and leucovorin (LV) in the treatment of patients with advanced breast cancer (ABC). Vinorelbine 25 mg m^−2^ 30-min intravenous infusion, and high-dose 5-FU 2600 mg m^−2^ plus LV 300 mg m^−2^ 24-h intravenous infusion (HDFL regimen) were given on days 1 and 8 every 3 weeks. Between June 1999 and April 2003, 40 patients with histologically confirmed recurrent or metastatic breast cancer were enrolled with a median age of 49 years (range: 36–68). A total of 25 patients had recurrent ABC, and 15 patients had primary metastatic diseases. The overall response rate for the intent-to-treat group was 70.0% (95% CI: 54–84%) with eight complete responses and 20 partial responses. All 40 patients were evaluated for survival and toxicities. Among a total of 316 cycles of VNB–HDFL given (average: 7.9: range: 4–14 cycles per patient), the main toxicity was Gr3/4 leucopenia and Gr3/4 neutropenia in 57 (18.0%) and 120 (38.0%) cycles, respectively. Gr1/2 infection and Gr1/2 stomatitis were noted in five (1.6%) and 59 (18.7%) cycles, respectively. None of the patients developed Gr3/4 stomatitis or Gr3/4 infection. Gr2/3 and Gr1 hand–foot syndrome was noted in two (5.0%) and 23 (57.5%) patients, respectively. Gr1 sensory neuropathy developed in three patients. The median time to progression was 8.0 months (range: 3–25.5 months), and the median overall survival was 25.0 months with a follow-up of 5.5 to 45+ months. This VNB–HDFL regimen is a highly active yet well-tolerated first-line treatment for ABC.

Breast cancer is the most common malignancy worldwide and is the fifth cancer killer in Taiwanese women. Despite adequate primary treatment with surgical resection and adjuvant chemohormonal therapy, about 25–30 and 75–80% of patients without and with axillary node involvement at mastectomy, respectively, will have recurrent and/or metastatic breast cancer within 10 years of surgery (EBCTCG, 1992a, 1992b, 1998; Horton, 1996). Combination chemotherapy will induce an objective response in approximately two-thirds of patients previously unexposed to chemotherapy, but complete eradication of disease at all sites will occur in less than 20% (Ross *et al*, 1985; Clavel and Catimel, 1993).

Among the active chemotherapeutic agents in the treatment of breast cancer, alkylating agents, anthracyclines, and taxanes are the main components of most standard regimens. Anthracycline-based combinations represent a highly active cytotoxic chemotherapeutic approach to the treatment of advanced breast cancer in the pre-taxane era (Ross *et al*, 1985: Clavel and Catimel, 1993). They are capable of inducing objective responses in 40–70% of patients (Ross *et al*, 1985; Clavel and Catimel, 1993). Further, taxane-based combinations also become major regimens for advanced breast cancer (Fornier *et al*, 2001; Buzdar *et al*, 2002; Davidson, 2002; Henderson *et al*, 2003). Among the active agents for advanced breast cancer, vinorelbine is also highly active both as a single agent and in combination regimens (Krikorian and Breillout, 1991).

Vinorelbine, a new semisynthetic vinca alkaloid, is well tolerated with significantly less neurotoxicity than other vinca alkaloids (vincristine and vinblastine) and a low incidence of subjective toxicities (Krikorian and Breillout, 1991). Vinorelbine, administered weekly as a single agent by an intravenous route, resulted in major objective response in about 45% of patients. Even in patients previously exposed to standard chemotherapy, 20–30% achieved a major objective response (Abeloff, 1995; Fumoleau *et al*, 1995; Hortobagyi, 1995; Smith, 1995). It is anticipated that vinorelbine will be increasingly utilised for treating metastatic breast cancer due to its favourable safety profile, good tolerability, and promising results in combination with other chemotherapy agents.

A phase I study suggested that the dose-limiting toxicity of vinorelbine was leucopenia and primarily neutropenia, and the maximum tolerated dose in single-agent therapy was 35.4 mg m^−2^ weekly (Mathe and Reizenstein, 1985). A series of phase II studies have shown that vinorelbine is a highly effective agent in the treatment of advanced breast cancer (Canobbio *et al*, 1989; Fumoleau *et al*, 1993; Garcia-Conde *et al*, 1994; Bruno *et al*, 1995). As first-line chemotherapy of advanced breast cancer, vinorelbine was studied in 4 phase II studies including 258 patients at a dose of 30 mg m^−2^ weekly (Canobbio *et al*, 1989; Fumoleau *et al*, 1993; Garcia-Conde *et al*, 1994; Bruno *et al*, 1995). The overall response rate was between 44% and 60%. The median duration of response was between 17.9 and 36 weeks, and median survival ranged from 50.3 to 73 weeks.

Vinorelbine was also used in combination with other chemotherapy agents in first-line treatment for advanced breast cancer. In phase II studies, the combination of doxorubicin (50 mg m^−2^ per cycle) (Spielmann *et al*, 1994) or epirubicin (90 mg m^−2^ per cycle) (Baldini *et al*, 1998) with 25 mg m^−2^ of vinorelbine on days 1 and 8 every 3 weeks resulted in a very impressive activity with an overall response rate of 74% (21% complete response plus 53% partial response) (Spielmann *et al*, 1994) and 70.2% (8.5% complete response plus 61.7% partial response) (Baldini *et al*, 1998), respectively. However, dose-limiting grade 4 neutropenia (70%) (Baldini *et al*, 1998) and treatment-related grade 2–4 cardiotoxicity (10%) (Spielmann *et al*, 1994) limited the clinical use of these combination regimens.

Searching for an effective yet well-tolerated regimen is mandatory. Among them, 5-fluorouracil (5-FU) is a potentially relevant candidate agent. 5-Fluorouracil could inhibit thymidylate synthase (TS) by 5-fluoro-2′-deoxyuridine monophosphate (FdUMP), a major metabolite of 5-FU, which binds to TS and prevents the formation of thymidine monophosphate (dTMP), which is an important precursor of thymidine triphosphate (dTTP), one of the four deoxynucleotides required for DNA synthesis (Grem *et al*, 1987; Bertino, 1997). Biochemical modulation of 5-FU by the addition of leucovorin (LV) results in the stabilisation of a ternary complex among FdUMP, LV, and TS. This enhances the inhibition of TS, the target enzyme of 5-FU (Grem *et al*, 1987; Bertino, 1997). Biochemical modulation of 5-FU by LV has been shown to have a better response than 5-FU alone in colorectal cancer (ACCMAP, 1992). A similar modulation effect of LV on 5-FU has also been shown in breast cancer (Fine *et al*, 1994).

In advanced breast cancer, in a phase II study of vinorelbine administered at an intended dose of 30 mg m^−2^ on days 1 and 5 in combination with 5-FU continuous infusion (750 mg m^−2^ daily for 5 days) every 3 weeks, an overall response rate of 61.6% was observed (Dieras *et al*, 1996a). The main toxicities (grades 3 and 4) were neutropenia (90% of patients), infection (12.7%), and mucositis (37%). The median response duration and overall survival were 12.3 and 23 months, respectively. Nole *et al* (1997) confirmed similar data in combination with vinorelbine, 5-FU, and LV with a response rate of 62%. However, the 5-day loading schedule of 5-FU (50 mg m^−2^ per day for 5 consecutive days as a continuous infusion) had significant treatment-related toxicity (such as 90% grade 3/4 neutropenia and 37% grade 3/4 mucositis, respectively), which may limit its clinical use (Dieras *et al*, 1996a). Searching for an effective yet well-tolerated combination is still mandatory.

Previously, we demonstrated that weekly 24-h infusion of high-dose 5-FU and LV (folinic acid), the HDFL regimen originally described by Ardalan *et al* (1991), appears to be particularly useful in gastric cancer (Hsu *et al*, 1997; Yeh and Cheng, 1998) and colon cancer (Yeh *et al*, 1997). We have provided evidence that prolonged exposure of gastric cancer cells to low concentration 5-FU for 24 h enhances the inhibition of TS, and thereby increases the cytotoxicity of 5-FU (Yeh *et al*, 2000b). Further, an HDFL regimen has repeatedly been demonstrated to cause minimal myelosuppression and therefore is an ideal component for combination chemotherapy with other cytotoxic agents against gastric cancer (Hsu *et al*, 1997; Yeh *et al*, 1997; Yeh and Cheng, 1998). We have also reported on the underlying mechanism of the low myelotoxicity of HDFL (Yeh *et al*, 2000a).

In one of our pilot studies, we used an HDFL regimen for the treatment of advanced breast cancer patients with heavily pretreated status or recurrence after high-dose chemotherapy with peripheral stem cells support. Even in this group of patients, an impressive response rate of 33% was noted.

In this study, we investigated if combination of weekly vinorelbine and weekly HDFL could be an effective yet well-tolerated regimen of the first-line treatment for patients with advanced breast cancer.

## PATIENTS AND METHODS

### Patients

The chemotherapy was used as the first-line therapy, while previous postmastectomy adjuvant therapy (e.g., CMF regimens, anthracycline (doxorubicin, epirubicin)-based regimens, or lower dose 5-FU (⩽750 mg m^−2^ per week)) given beyond 6 months before study enrolment was acceptable.

Eligibility criteria included (1) pathologically confirmed, recurrent or metastatic breast cancer, (2) at least one bidimensional measurable lesion on imaging studies, (3) ECOG performance status ⩽2, (4) age between 18 and 75 years, (5) adequate hepatic, renal, and bone marrow functions, and (6) fasting serum TG (1 day before the first cycle of chemotherapy) >70 mg dl^−1^. The lower limit for serum TG was set to avoid HDFL-related hyperammonemic encephalopathy, which occurs in around 5% of Taiwanese patients (Yeh and Cheng, 1997). Fasting serum TG level ⩽70 mg dl^−1^ is the most important risk factor for HDFL-related hyperammonemic encephalopathy.

Exclusion criteria included (1) previous treatment with high-dose 5-FU (⩾2000 mg m^−2^ per week), (2) prestudy fasting serum TG level ⩽70 mg dl^−1^, (3) pregnant, breast-feeding, or woman of child-bearing potential without adequate contraception, (4) patients who refused placement of a central venous indwelling catheter (Port-A catheter) for outpatient chemotherapy, (4) concurrent or prior malignancy except curatively resected cervical carcinoma *in situ* or squamous cell carcinoma of skin, (5) central nervous system metastases, (6) active infection, and (7) concurrent treatments that could interfere with the study evaluation. This study was approved by the ethics committee of National Taiwan University Hospital and Changhua Christian Hospital. Signed informed consent was obtained from all patients.

### Study design

This was a prospective phase II clinical trial.

### Chemotherapy protocol

On days 1 and 8 of each cycle of chemotherapy, vinorelbine 25 mg m^−2^ was given as a 30-min intravenous infusion, and followed by 5-FU 2600 mg m^−2^ and LV (folinic acid) 300 mg m^−2^ given as a continuous 24-h intravenous infusion. Treatment was repeated every 21 days. Treatment consisted of at least two cycles unless rapid disease progression occurred during treatment. Response assessment was performed every two cycles. Patients with complete response, partial response or stable disease (SD) continued the protocol treatment until intolerable toxicity. Patients with progressive disease were removed from the protocol treatment, and received other salvage treatment under the discretion of the responsible physicians. The response rate reported was the best tumour response obtained by the study treatment. Tumour response to the salvage therapy has not been included in the evaluation of the response rate.

### Dose modification

For the first cycle of vinorelbine–HDFL, the following criteria were necessary: WBC count ⩾4000 *μ*l^−1^ or ANC ⩾2000 *μ*l^−1^, platelet count ⩾100 000 *μ*l^−1^, serum creatinine ⩽1.5 mg dl^−1^, normal serum bilirubin level, and transaminases (AST or ALT) ⩽3.5-fold of the upper normal limits (UNLs) of reference values.

Patients were treated with the next cycle of vinorelbine–HDFL on day 22 or within a 3-week interval from the previous cycle of vinorelbine–HDFL. No dose reduction for vinorelbine was allowed. However, in cases of haematological, hepatic, or neurological toxicity, schedule modification was recommended as follows.

### Haematological toxicity

Schedule modification for vinorelbine was based on blood count results obtained within 2 days of treatment, according to the following schedule: if leucopenia and/or thrombocytopenia ⩽grade 2 (WBC count ⩾2000 *μ*l^−1^ and platelet count ⩾50 000 *μ*l^−1^), no schedule delay was indicated; and if leucopenia or thrombocytopenia ⩾grade 3 (WBC count <2000 *μ*l^−1^ or platelet count <50 000 *μ*l^−1^), schedule delay and reassessment were indicated. If the study treatment could not be administered after a 3-week interval because of haematological toxicity, the patient was removed from protocol treatment.

### Neurological toxicity

If peripheral neuropathy ⩾grade 2, schedule delay and reassessment were indicated. If the study treatment could not be administered after a 3-week interval because of neurological toxicity, the patient was removed from protocol treatment.

Patients with pretreatment (before the first cycle of vinorelbine–HDFL) fasting serum TG level ⩽70 mg dl^−1^ were not neligible for this trial (Yeh and Cheng, 1997). If patients developed ⩾grade 2 toxicity of state of consciousness, HDFL was discontinued immediately until complete recovery was achieved under the best supportive care (Yeh and Cheng, 1997). Upon the altered state of consciousness, plasma ammonia level, lactic acid level, arterial blood gas, and complete biochemical screening (including the TG level) were immediately checked and the extent of HDFL-related neurotoxicity was documented. In the subsequent cycles, a 40% dose reduction for both 5-FU and LV was indicated.

### Hepatic toxicity

If total bilirubin or AST/ALT levels were abnormal in the absence of progressive disease, the following dose modifications were applied to vinorelbine: (1) ⩽grade 2 toxicity, that is, total bilirubin or AST/ALT was 1.26- to 5.0-fold of the UNLs of reference values, no dose modification was indicated, (2) grade 3 toxicity, that is, total bilirubin or AST/ALT was 5.1- to 10.0-fold of the UNLs, schedule delay and reassessment were indicated; and (3) grade 4 toxicity, that is, total bilirubin or AST/ALT was more than 10.0-fold of the UNLs, vinorelbine was discontinued in the subsequent cycles.

### Other toxicities

When ⩾grade 3 diarrhoea or stomatitis developed, HDFL chemotherapy was temporarily stopped for schedule delay and was reused after diarrhoea and stomatitis subsided to ⩽grade 1.

### Evaluation of efficacy and toxicities

Evaluations before chemotherapy included medical history taking, physical examination, complete blood count, blood chemistry, chest X-ray, and computed tomography (CT) scan of chest or abdomen as indicators of the location of lesions. After starting protocol treatment, complete blood count was examined weekly and blood chemistry every 2 weeks. The patients' condition and treatment-related toxicities were evaluated weekly. Tumour size was measured by imaging studies every two cycles, or when there were any clinical signs of possible tumour progression. Tumour response was evaluated according to the World Health Organization (WHO) criteria. Toxicities were graded using the NCI-common toxicity criteria (version 2.0).

### Statistical methods

This was a phase II, two-institutional, prospective study. The Simon two-stage design was used. The response rates of interest were *P*_0_=50% and *P*_1_=70%. If there were more than 11 responders of the 23 patients in the first stage, the study then continued to enrol a total of 40 patients. If there were more than 24 responders of the 40 patients, this treatment would be acceptable with *α* of 0.10 and *β* of 0.10. Patients evaluable for response were those who had finished at least two cycles of treatment. All enrolled patients were subjected to toxicity evaluation.

Time to progression was defined as the duration from the date of starting protocol treatment to the date of documented disease progression or death by any cause. Overall survival was defined as the duration from the date of starting protocol treatment to the date of death. The Kaplan–Meier method was used in all survival analyses.

## RESULTS

### Patients and treatment

Between June 1999 and April 2003, 40 patients were enrolled in the study. The major clinicopathologic features of the patients are listed in Table 1. The median age was 49 years (range: 36–68). A total of 25 patients had recurrent advanced breast cancer (15 had prior anthracycline-containing adjuvant chemotherapy, two had CMF adjuvant chemotherapy), and 15 patients had primary metastatic diseases. A total of 10 (25%) patients had bone/soft tissue disease, and 10 (25%) patients had three or more sites of metastatic diseases. Eight (20%) patients had had postmastectomy adjuvant chemotherapy for more than 2 years; although they were not chemonaive patients, the study regimens for them were the first-line treatment after metastasis or recurrence.

A total of 316 cycles (median: 8; mean: 7.9; range: 4–14) of chemotherapy were given. In all, 107 (33.9%) cycles needed a median schedule delay of 7 days (range: 3–14 days) mainly due to grade 3/4 neutropenia.

### Efficacy

All 40 patients were evaluated for response. There were 16 responses in 23 patients in the first stage. Therefore, the study continued to enrol a total of 40 patients as scheduled by the two-stage design. None of the patients had early progression or excluded from analysis for tumour response. There were eight patients with complete remission (CR), 20 patients with partial remission (PR), and 12 patients with SD. The overall response rate for the intent-to-treat group was 70.0% (95% confidence interval: 54–84%). The median time to tumour response was 1.5 months (range: 1.5–3.5 months). Four patients (10%) dropped out after completion of two, four, six, and 12 cycles of study treatment in response conditions (one CR and three PR) due to treatment refusal. All of them were included in the survival analysis. None of the enrolled patients lost to follow-up.

**Figure 1:**
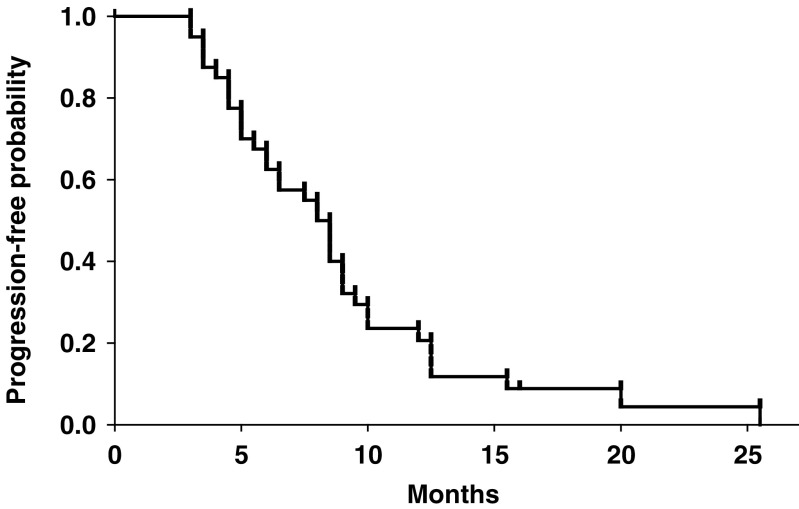
Kaplan–Meier curve for time-to-progression of the 40 patients.

**Figure 2:**
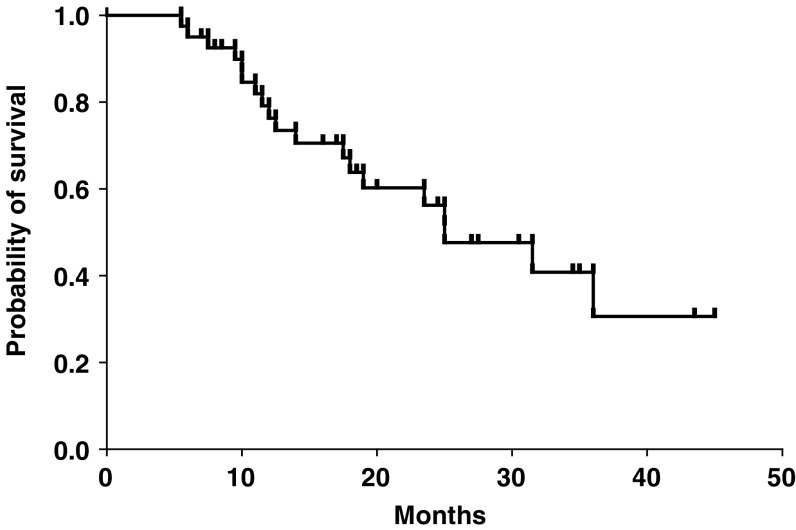
Kaplan–Meier curve for overall survival of the 40 patients.

Salvage therapy after disease progression included anthracycline-containing regimens, taxane-based regimens, trastuzumab, and aromatase inhibitors (anastrozole or letrozole) in nine, 19, six, and 11 patients, respectively. The median follow-up time of the whole group of 40 patients was 27 months. The median time to progression was 8.0 months (range: 3–25.5 months) (Figure 1). The median overall survival was 25 months (range: 5.5 to 45+ months) (Figure 2).

### Toxicity

All 40 patients were evaluated for toxicities (Table 2). The main toxicity was grade 3/4 leucopenia and grade 3/4 neutropenia in 57 (18.0%) and 120 (38.0%) cycles, respectively. Grade 1/2 infection and grade 1/2 stomatitis were noted in five (1.6%) and 59 (18.7%) cycles, respectively. None of the patients developed grade 3/4 stomatitis or grade 3/4 infection. Grade 3/4 and grade 1/2 diarrhoea was noted in 1 (0.3%) and 26 (8.2%) cycles, respectively. Grade 2/3 and grade 1 hand–foot syndrome was noted in two (5.0%) and 23 (57.5%) patients, respectively. One patient needed a 25% dose reduction of 5-FU due to grade 4 diarrhea, and one patient needed a 40% dose reduction of 5-FU/LV due to hyperammonemic encephalopathy during the first cycle. Grade 1 sensory neurotoxicity developed in three patients. Other nonhaematological toxicities were negligible. No patients discontinued protocol treatment because of vinorelbine-related neuropathy. Due to cautious prestudy screening of fasting serum TG level and meticulous guidelines for dose modification of 5-FU and LV, only one of the patients developed HDFL-related hyperammonemic encephalopathy (Yeh and Cheng, 1997).

## DISCUSSION

The results of this phase II study indicated that this vinorelbine–HDFL regimen using weekly vinorelbine and weekly 24-h infusion of high-dose 5-FU/LV is effective for the treatment of advanced breast cancer. The overall response rate of 70.0% (95% confidence interval: 54–84%) was within the range (40–70%) of previously reported major protocols of anthracycline-based regimens (Ross *et al*, 1985; Clavel and Catimel, 1993; Jassem *et al*, 2001; Biganzoli *et al*, 2002; Sledge *et al*, 2003), taxane-based regimens (Jassem *et al*, 2001; Biganzoli *et al*, 2002; Sledge *et al*, 2003), and anthracycline-and-taxane-based regimens (Razis and Fountzilas, 2001; Sledge *et al*, 2003). Previously, studies from other series using vinorelbine and different administration schedules of 5-FU and LV have also demonstrated a good efficacy in breast cancer (Dieras *et al*, 1996a; Nole *et al*, 1997). However, the 5-day loading schedule of 5-FU created significant treatment-related toxicity, which may limit its clinical use (Dieras *et al*, 1996a). We report that this vinorelbine–HDFL regimen using a unique schedule of 5-FU and LV is an effective yet well-tolerated combination for advanced breast cancer.

In this study, 5-FU and LV were given in two doses of weekly 24-h infusion (HDFL regimen) every 3 weeks. The rationale for this scheduling of 5-FU/LV was based on our previous studies, which indicated that HDFL is in general a highly effective and very safe regimen for advanced gastric cancer (Hsu *et al*, 1997; Yeh and Cheng, 1998) and colorectal cancer (Yeh *et al*, 1997). In our pilot studies, we used an HDFL regimen for the treatment of advanced breast cancer patients with heavily pretreated status or recurrence after high-dose chemotherapy. Even in this group of patients, an impressive response rate of 33% was noted.

The patients' compliance with this HDFL regimen was generally good. Further, results of our *in vitro* studies have implied that strict avoidance of bolus injection of 5-FU is the key to avoiding myelosuppression (Yeh *et al*, 2000a). Although the best protocol of 5-FU remains to be explored (Bertino, 1997; Grem, 2001), both clinical and laboratory data indicate that HDFL is an ideal component for combination chemotherapy with other cytotoxic agents. For instance, paclitaxel followed by weekly high-dose 5-FU and LV infusion has been shown to have a 55% response rate in anthracycline-resistant metastatic breast cancer patients (Klaassen *et al*, 1995, 1996). In this study, we showed that weekly vinorelbine plus weekly HDFL is an effective regimen with well-tolerated toxicities for the first-line treatment of advanced breast cancer.

Although survival is not a major end point for evaluation of efficacy in phase II trials, the median overall survival of 25 months in this study was favourably within the range of other previously reported major protocols of anthracycline-based regimens (Ross *et al*, 1985; Clavel and Catimel, 1993; Jassem *et al*, 2001; Biganzoli *et al*, 2002; Sledge *et al*, 2003), taxane-based regimens (Jassem *et al*, 2001; Biganzoli *et al*, 2002; Sledge *et al*, 2003), and vinorelbine-based regimens (Dieras *et al*, 1996a; Nole *et al*, 1997; Norris *et al*, 2000).

The toxicity of the current vinorelbine–HDFL protocol is generally well tolerated. Although vinorelbine-related neurotoxicity did occur in this study, it was usually mild (grade 1/2) under this weekly schedule. No patients discontinued protocol treatment due to vinorelbine-related neuropathy. In contrast, the paclitaxel-related neuropathy was found to be one of the dose-limiting toxicities in paclitaxel-containing regimens for breast cancer (Postma *et al*, 1995; Forsyth *et al*, 1997; Gelmon *et al*, 1999). Other major toxicities, including diarrohea and stomatitis, were of lesser severity than for other major regimens using vinorelbine and a 5-day schedule of 5-FU and LV (Dieras *et al*, 1996a; Nole *et al*, 1997). Neutropenia and leucopenia were not less severe than other reported regimens using vinorelbine and 5-FU; however, the doses, schedules, and G-CSF support of these regimens were essentially different from the current regimen (Kornek *et al*, 1998; Lombardi *et al*, 2000). Regimen used by Kornek *et al* (1998) required G-CSF support at 5 *μ*g kg^−1^ day^−1^ subcutaneously on days 6–10 during each cycle, and it was complicated with septicaemia in two patients. Regimen used by Lombardi *et al* (2000) applied lower dose intensity of vinorelbine (20 mg m^−2^ on days 1 and 8, every 4 weeks) than current regimen (25 mg m^−2^ on days 1 and 8, every 3 weeks). In addition, the regimen by Lombardi *et al* (2000) used protracted continuous infusion (PCI) of low-dose 5-FU (250 mg m^−2^ day^−1^) (Lombardi *et al*, 2000), rather than a convenient weekly HDFL. Although grade 3/4 neutropenia and grade 3/4 leucopenia were still common, the rarity of both grade 3/4 stomatitis and grade 3/4 diarrohea may contribute to the absence of grade 3/4 infections in this study. Prophylactic G-CSF support was not used in this study. A total of 117 (33.9%) cycles needed a median delay of 7 days (range: 3–14 days). Prophylactic antibiotics with oral quinolones were used in 7.5% of patients. Overall, the toxicity profiles of the current vinorelbine–HDFL protocol were generally well tolerated.

We conclude that combination of weekly vinorelbine and weekly 24-h infusion of high-dose 5-FU and LV is a highly effective regimen with well-tolerated toxicities for the first-line treatment of advanced breast cancer.

## Figures and Tables

**Table 1:** Clinicopathologic features of the patients

	**Patient number**
Total patients	40
Age, median (range)	49 (36–68)
ECOG performance:	
0	10 (25.0%)
1	24 (60.0%)
2	6 (15.0%)
Menopausal status	
Premenopause	18 (45.0%)
Postmenopause	22 (55.0%)
Oestrogen receptor status	
Positive	21 (52.5%)
Negative	11 (27.5%)
Unknown	8 (20.0%)
Disease status	
Recurrence/metastasis	25 (62.5%)
*de novo* metastasis	15 (37.5%)
Prior therapy	
Anthracycline-containing adjuvant chemotherapy	15 (37.5%)
CMF adjuvant chemotherapy	2 (5.0%)
Hormonal therapy	21 (52.5%)
Local radiotherapy	11 (27.5%)
Disease sites	
Lymph nodes	33 (82.5%)
Lung	26 (65.0%)
Breast	19 (47.5%)
Bone or/and spine	16 (40.0%)
Liver	12 (30.0%)
Pleural effusion	8 (20.0%)
Skin	6 (15.0%)
Others	1 (2.5%)

**Table 2:** Toxicity of the vinorelbine–HDFL regimen

**Toxicity**	**Patients (*n*=40)**		**Cycles (*n*=316)**	
	**Grade 1–2**	**Grade 3–4**	**Grade 1–2**	**Grade 3–4**
Haematological				
Neutropenia	17.5%	80.0%	37.7%	38.0%
Leucopenia	47.5%	40.0%	48.2%	18.0%
Thrombocytopenia	10.0%	0	2.5%	0
Febrile neutropenia	—	2.5%	—	0.3%
Gastrointestinal				
Nausea	80.0%	5.0%	49.4%	1.6%
Vomiting	47.5%	10.0%	18.4%	2.2%
Diarrhoea	35.0%	2.5%	8.2%	0.3%
Stomatitis	55.0%	0	18.7%	0
Neuropathy	7.5%	0	3.8%	0
Hepatic	67.5%	2.5%	28.8%	0.3%
Others				
Hand–foot syndrome	57.5%^a^	5.0%^b^	NA	NA
Infection	5.0%	0	1.6%	0
Alopecia	75.0%	2.5%	54.7%	0.6%

a Grade 1.

b Grade 2–3.

NA=nonapplicable by cycles.
